# Kenneth Charles Holmes 1934–2021

**DOI:** 10.3389/fmolb.2022.855014

**Published:** 2022-03-18

**Authors:** Belinda Bullard

**Affiliations:** Department of Biology, University of York, York, United Kingdom

**Keywords:** fiber diffraction, crystallography, muscle, cryo-EM, actin, synchrotron radiation

Ken Holmes was a versatile experimentalist. He applied his knowledge of the physical sciences to developing methods for determining the structure of biological samples. The need for more intense X-rays for the fiber diffraction of an insect muscle led to his outstanding contribution to structural biology: the use of synchrotron radiation as a source of X-rays for determining biological structures. This revolutionized protein crystallography, resulting in the determination of the structure of hundreds of thousands of proteins. Ken was also a good communicator. Many will remember his enthusiastic descriptions of “How muscle works,” even if they were not the definitive solution.

Ken read physics at St John’s College, Cambridge, obtaining a BA in 1955. He then moved to Birkbeck College in London, where he worked with Rosalind Franklin and later with Aaron Klug on the structure of the tobacco mosaic virus (TMV). TMV was known to be rod-shaped with protein subunits arranged helically around the long axis of the rod; there was also an RNA component. Ken and Franklin obtained X-ray fiber diagrams of oriented gels of the virus in a capillary using a finely focused X-ray camera. Diffraction patterns from samples with a single mercury atom on each protein subunit enabled them to determine the symmetry parameters of TMV (Franklin and Holmes, 1956, 1958). The isomorphous replacement method also revealed that a single strand of RNA is inside the virus and that there is central hollow core. Ken received a PhD for this work in 1959. The technique of X-ray fiber diffraction learnt from Franklin was to influence Ken’s future career.

In 1960, Ken moved to the Children’s Hospital in Boston to continue the work on TMV with a post-doctoral position in Don Caspar’s lab. Ken perfected the preparation of virus particles, and the large amounts of data were processed on an early IBM machine (Caspar and Holmes, 1969). Carolyn Cohen was also at the Children’s Hospital working on the structure of muscle proteins. The anterior byssus retractor muscle (ABRM) of *Mytilus edulis* was particularly interesting because it went into a “catch” state to keep the two halves of the shell shut. Ken was able to squeeze the ABRM into a capillary, with a technique similar to the one he used with TMV, forming an oriented gel. This gave very good X-ray fiber diffraction patterns, which Ken and Cohen showed were consistent with a coiled coil α-helical structure in paramyosin in the core of the myosin filaments (Cohen and Holmes, 1963). Although this introduction to muscle research was a sideline, it became the chief interest for most of Ken’s career.

Back in England in 1962, Ken moved to the Laboratory of Molecular Biology (LMB) in Cambridge to continue work on TMV in Aaron Klug’s group. During his early years at LMB, Ken developed a rotating anode X-ray generator with Bill Longley in order to improve the fine focus needed for the TMV work. This was a prototype of the Elliott X-ray generator. Meanwhile, John Pringle had become Linacre Professor of Zoology in Oxford in 1961. He was interested in the mechanism of oscillatory contraction of insect flight muscle. Richard Tregear joined him and set out to measure changes in the X-ray fiber diagram during the cyclical contraction of the flight muscle. Pringle had introduced the giant water bug, *Lethocerus*, as a model system for work on the flight muscle. The muscle fibers are up to 1 cm long, easily separated, and ideal for mechanical measurements. Tregear visited Cambridge to get advice from Ken about setting up an Elliott rotating anode in Oxford. He brought the flight muscle from the largest *Lethocerus* species, *Lethocerus maximus*. They used the Holmes-Longley fine focus rotating anode X-ray generator and an X-ray camera with a gold-plated glass mirror and bent quartz crystal monochromator (developed by Ken and Hugh Huxley) to take low-angle pictures of fibers in rigor and fibers relaxed by adding adenosine 5’-triphosphate (ATP) to the solution. There was a strong meridional reflection at 14.5 nm in the relaxed fibers that was absent when the fibers were in rigor. Mike Reedy was a post-doc with Huxley. He described a chance meeting with Ken and Tregear, who were uncertain about how to interpret the changes in the X-ray reflections. Reedy fixed the flight muscle fibers while they were in the X-ray beam. He embedded the fibers in the rigor or relaxed state and cut thin sections for electron microscopy. This showed that crossbridges spaced at 14.5 nm on thick filaments containing myosin were perpendicular to the long axis of the relaxed fiber and at an angle of 45° when fibers were in rigor. This was evidence for the swinging crossbridge model (Reedy et al., 1965). The careers of Ken and Reedy were immediately affected by this striking result. David Phillips, who was the professor of Molecular Biophysics in Oxford, offered Ken a position in the new department. This was supported by Pringle, who wanted to expand muscle research in the Zoology department, which then housed Biophysics. Ken turned down the position, preferring to accept the offer to set up a Biophysics department in the Max Planck Institute in Medical Research in Heidelberg. He thought that this would give him greater autonomy. Reedy became an assistant professor at UCLA.

In 1968, Ken resumed work on the structure of TMV in Heidelberg. He and collaborators made several heavy atom derivatives of the protein and determined the structure to 4Å resolution, including the structure of the RNA and its binding site (Holmes et al., 1972, 1975). Ken now left TMV to others and turned back to the muscle. He and Huxley had come to an agreement that Huxley would work on frog muscle and Ken on insect muscle. They aimed to record changes in fiber diffraction during contractions. In 1970, Gerd Rosenbaum and Ken established that the synchrotron at DESY Hamburg produced intense X-rays suitable for obtaining fiber diagrams from insect muscle. By 1972, Ken with Rosenbaum and John Barrington-Leigh had constructed the first X-ray beamline at DESY. Improvements were made by Ken and his team, including introduction of a quartz monochromator to improve the focus of the X-ray beam. The first measurements were made with *Lethocerus* flight muscle (Rosenbaum et al., 1971; Goody et al., 1975). The potential of the X-ray beam at DESY for solving biological structures led to the facility being transferred to EMBL in 1975 to become an EMBL Outstation. As Ken put it: "The need to record low-angle scattering x-ray fibre diagrams from muscle with milli-second time resolution drove the use of synchrotron radiation as an x-ray light source. The first smudgy diffraction patterns were obtained from a slice of insect flight muscle. Out of this grew the EMBL Outstation at DESY" (Holmes and Rosenbaum, 1998). Later, the storage ring, DORIS, which generated X-rays of greater intensity, was used for protein crystallography and fiber diffraction at Hamburg. There followed a worldwide expansion in the determination of protein structures using synchrotron radiation as an X-ray source.

Ken now turned to the structure of actin using the technique of X-ray fiber diffraction with oriented gels of polymerized actin in a capillary. Valerie Lednev, a visiting Russian scientist, and David Popp obtained good fiber diffraction of actin gels using a rotating anode X-ray source (Popp et al., 1987). However, the structure of the actin monomer was needed to fit to the monomer structure in the filament. This was achieved when Wolfgang Kabsch, Dietrich Suck, and others solved the structure of an actin-DNase 1 complex (Kabsch et al., 1990); they were able to do this because Uno Linberg had previously shown that DNase 1 binds to actin and prevents actin forming filaments. Ken and his co-workers obtained a fit of the actin monomer in the filament; this was refined by Michael Lorenz and the improved model was widely used for many years (Holmes et al., 1990; Lorenz et al., 1993). Ken with Lorenz, Rosenbaum, and others extended the fiber diffraction of oriented actin gels to gels of an actin-tropomyosin complex. The resulting model showed each tropomyosin pseudo-repeat interacting with an actin monomer in the same way, although there was no direct contact between tropomyosin and actin; the model was in agreement with images obtained by electron microscopy (Lorenz et al., 1995). It was not until 2009 that Toshiri Oda, Yuchiro Maeda, and others, using better oriented actin gels and a synchrotron X-ray source, showed that a domain of the actin monomer in the filament is rotated relative to its position in unpolymerized actin (Oda et al., 2009). Ken returned to studying the actin-tropomyosin complex using the new structure for the actin filament and a curved structure for tropomyosin derived from electron micrographs. Ken, with Stefan Fischer and Bill Lehman’s group, modeled tropomyosin as a relatively rigid structure with bends allowing the molecule to follow the actin helix (Li et al., 2010).

When the crystal structure of the actin-binding head region of myosin (S1) was determined in 1993, Ken started to model the binding of S1 to the actin filament; this was to be the main focus during the next stage of his career. Images of acto-S1 were obtained by cryo-electron microscopy. By fitting the structure of S1 in different nucleotide states to the structure of the acto-S1 complex, Ken and colleagues produced a model in which the angle of the lever arm in the distal part of S1 changed during the power stroke, while the angle of the actin-binding region remained constant (Rayment et al., 1993; Schröder et al., 1993; Holmes et al., 2003, 2004). The model suggested by Ken and colleagues for generation of the power stroke is fundamentally similar to current models: ATP binds to S1 and is hydrolyzed, S1 binds to actin with the lever arm up and an actin-binding cleft in S1 open (the pre-power stroke), phosphate migrates from the active site, the actin-binding cleft closes and the lever arm moves down to produce the power stroke of about 11 nm. Release of adenosine diphosphate (ADP) follows, resulting in the rigor state, corresponding to the end of the power stroke, first observed by Reedy, Holmes, and Tregear in 1965. The early model continues to be refined by others as more crystal structures of S1 using synchrotron X-ray sources became available, together with higher-resolution structures of acto-S1 determined by cryo-electron microscopy (Houdusse and Sweeney 2016; Robert-Paganin et al., 2020). Ken’s career saw the solution to the problem of how muscle works evolve from the first fiber diffraction patterns to the crystallographic structures of actin and myosin S1 and today to the high-resolution structure of the muscle sarcomere obtained using cryo-electron microscopy by Stefan Raunser and colleagues (Wang et al., 2021).

Ken and Klug both worked with Franklin at Birkbeck college. Franklin died before Ken had finished his PhD; Klug became the head of the group, and Ken learnt theoretical aspects of how to interpret fiber diffraction patterns from him. Their collaboration continued when the Birkbeck TMV group moved to LMB in Cambridge. Towards the end of his career, Ken embarked on a biography of Klug (Holmes, 2017). This meant frequent trips to Cambridge to talk to Klug and to visit the Archive Centre at Churchill College, which has the Klug papers (Figure). The book contains technical descriptions of Klug’s work on viruses and his analysis of electron microscopy images. It also gives a revealing picture of the academic life of molecular biologists in the UK at that time. During his visits to Cambridge, Ken re-established his links with St John’s College, even wearing the red boat club blazer from his undergraduate days to a college dinner.

**FIGURE:**
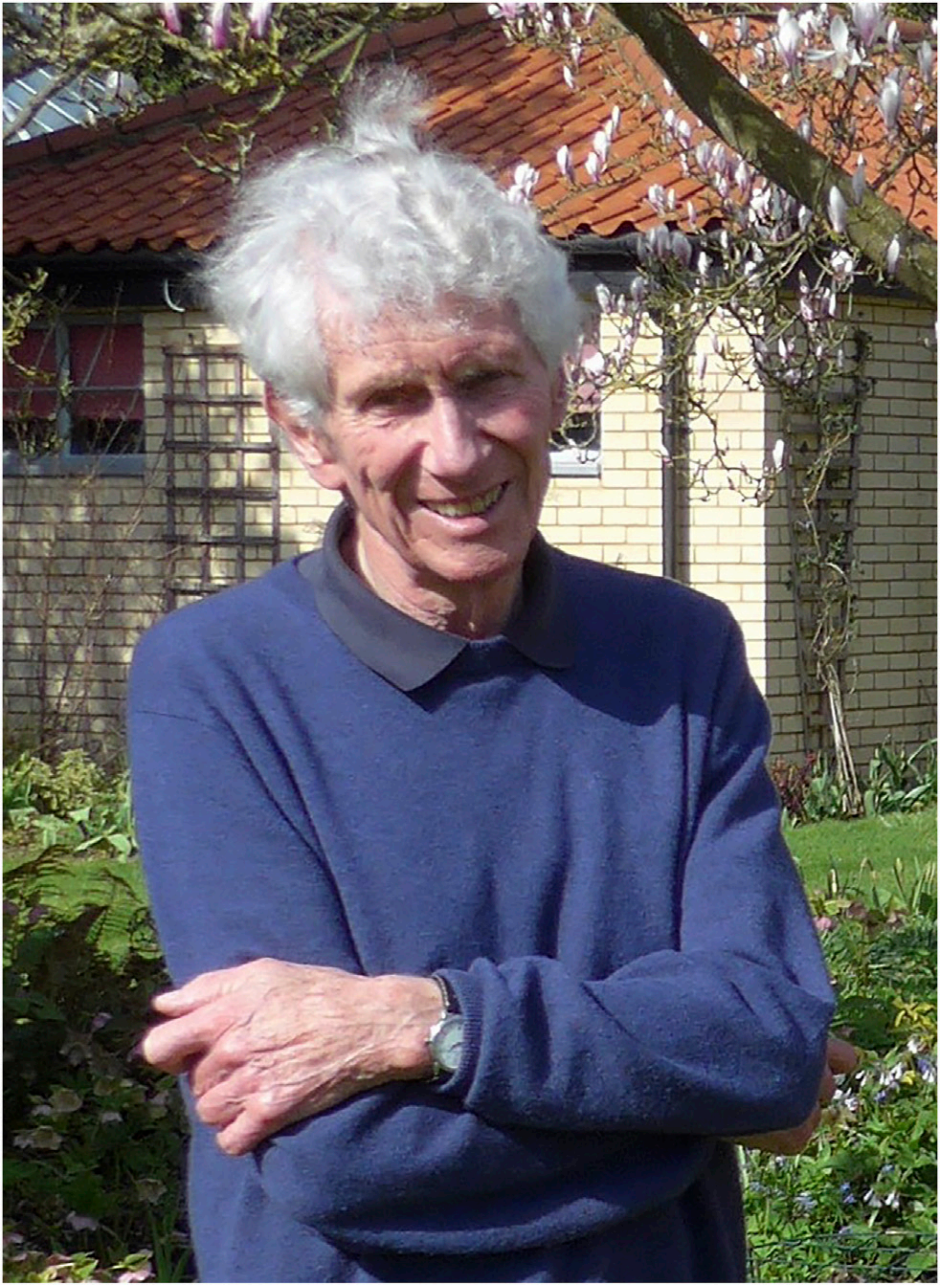
Ken on a visit to Cambridge in 2017.
